# Human Activity Prediction Based on Forecasted IMU Activity Signals by Sequence-to-Sequence Deep Neural Networks

**DOI:** 10.3390/s23146491

**Published:** 2023-07-18

**Authors:** Ismael Espinoza Jaramillo, Channabasava Chola, Jin-Gyun Jeong, Ji-Heon Oh, Hwanseok Jung, Jin-Hyuk Lee, Won Hee Lee, Tae-Seong Kim

**Affiliations:** 1 Department of Electronics and Information Convergence Engineering, Kyung Hee University, Yongin 17104, Republic of Korea; inespinoza24@khu.ac.kr (I.E.J.); channabasavac7@khu.ac.kr (C.C.); wjdwlsrbs77@khu.ac.kr (J.-G.J.); dhwlgjs3@khu.ac.kr (J.-H.O.); hwan136@khu.ac.kr (H.J.); qlqjs3647@khu.ac.kr (J.-H.L.); 2 Department of Software Convergence, Kyung Hee University, Yongin 17104, Republic of Korea; whlee@khu.ac.kr

**Keywords:** human activity prediction, inertial measurement unit, deep learning forecasting, sequence-to-sequence encoding, attention

## Abstract

Human Activity Recognition (HAR) has gained significant attention due to its broad range of applications, such as healthcare, industrial work safety, activity assistance, and driver monitoring. Most prior HAR systems are based on recorded sensor data (i.e., past information) recognizing human activities. In fact, HAR works based on future sensor data to predict human activities are rare. Human Activity Prediction (HAP) can benefit in multiple applications, such as fall detection or exercise routines, to prevent injuries. This work presents a novel HAP system based on forecasted activity data of Inertial Measurement Units (IMU). Our HAP system consists of a deep learning forecaster of IMU activity signals and a deep learning classifier to recognize future activities. Our deep learning forecaster model is based on a Sequence-to-Sequence structure with attention and positional encoding layers. Then, a pre-trained deep learning Bi-LSTM classifier is used to classify future activities based on the forecasted IMU data. We have tested our HAP system for five daily activities with two tri-axial IMU sensors. The forecasted signals show an average correlation of 91.6% to the actual measured signals of the five activities. The proposed HAP system achieves an average accuracy of 97.96% in predicting future activities.

## 1. Introduction

Human Activity Recognition (HAR) has garnered significant attention due to its potential to improve the quality of life in daily tasks by providing real-time monitoring and feedback across various fields. These applications range from activity and healthcare assistance [1,2], fitness [3], muscular rehabilitation [4], occupational safety [5], smart home monitoring [6], and driver monitoring [7]. For HAR, traditional machine learning techniques, such as support vector machines (SVM), K-Nearest Neighbors (KNN), and random forest trees [8,9], have been used in these applications. Deep learning models such as Convolutional and Recurrent Neural Networks (C/RNN) [10,11,12] have recently gained popularity for their superior feature extraction and recognition abilities. However, most HAR works are based on past sensor data. HAR works based on future data (i.e., activity prediction) are still rare [13], although activity prediction could be crucial in several real-world scenarios, such as fall predictions or sports routines, to prevent injuries. Prior HAP works can be categorized into two main kinds: video-based and sensor-based prediction. Most video-based HAP works rely on past activity video frames to predict future action frames and classify them to predict future activities. In [14,15], custom Generative Adversarial Networks (GANs) were used for early activity prediction. These works predicted future frames from the observed videos with a generator. They classified the predicted frames among a set of possible activities in indoor controlled environments. Meanwhile, in [16], a Long Short-Term Memory Networks (LSTM) model was used to obtain an anticipated action prediction using an egocentric camera mounted on the head of the subjects. In [17], a long-term prediction was achieved through a CNN-RNN model that first classified activities from the video and then predicted the future labels.

In sensor-based HAP works, recent systems have been proposed in both HAR and HAP to overcome the limitations of video-based works, including privacy concerns, object occlusion, and dependency on environmental contrast (i.e., lighting, fixed viewpoints, and object overlapping). These works are primarily based on Inertial Measurement Units (IMU) sensors, including accelerometers, gyroscopes, and magnetometers in wearable devices (i.e., smartwatches, rings, and sports straps). Although sensor-based HAR systems have been popular due to the growth in wearable devices in daily life [18,19], sensor-based HAP works are rare and have not been actively investigated. Recently, multivariate motion signal predictions from IMU sensors have been proposed through attention models, capable of retaining pattern and feature information across multiple channels [20,21,22,23,24]. Therefore, these forecasting models on time series data can be used in HAP to generate future activity signals, making HAP feasible by classifying the forecast signals. In [21], a CNN network mixed with a Fourier transform was proposed to convert time series data into 3D representation to predicted future signals obtaining a Mean Square (MSE) value of 0.134. The system was evaluated in a fall detection study, predicting a fall up to 0.6 s before the event. In [22], an adversarial network based on a transformer model and attention layers was used to forecast motion signals and predict the fatigue level during sports training routines. They obtained a Pearson correlation of 0.92 and a fatigue accuracy of 83%. Furthermore, forecasting and data augmentation models have been proposed using GAN frameworks in multiple time series applications such as financial, sensor signals, and weather predictions [25]. In [26], the SynSigGAN model was proposed using a GridBiLSTM module as the generator and a CNN module as the critic network. This model achieved an RMSE value of 0.25 in generating future data. Also, similar models to SynSigGAN, namely TimeGAN [27], NR-GAN [28], and RCGAN [29], have been proposed for multichannel data generation and forecasting. A recent attempt has included transformer encoders as the generator and critic network in TTS-GAN [30], obtaining better results than traditional GAN structures based on RNN or CNN models. However, these GAN-based approaches still present drawbacks during the training stage. For instance, these models are difficult to train and finetune, being sensitive to hyperparameters, making the models unstable during the training. Despite the recent approaches in multivariate data forecasting, multiclass human activity prediction based on multiple signal channels has not been investigated yet.

In this work, we present a multiclass HAP system employing a deep forecaster model to predict future activity signals and a deep classifier model to recognize future activities based on the forecasted data. For the deep forecaster, we propose an encoder–decoder network based on Sequence-to-Sequence (Seq2Seq) Long Short-Term Memory (LSTM) with Multi-head Attention (MA) and Positional Encoding (PE) layers. These attention mechanisms make it possible to preserve the spatial features and temporal dependencies across the IMU sensor channels. For the forecaster comparison, we have tested four extra models with time series data, including two based on classic deep learning networks, namely Conv2LSTM and Seq2Seq-LSTM, and two GAN-based models, namely SynSigGAN and TTS-GAN. For multiclass HAP, we have studied five daily activities (i.e., walk, run, Nordic walk, ascend stairs, and descend stairs) collected from six accelerometer channels of two IMU sensors located at the chest and ankle of five subjects. Once the forecasted data is obtained, we use a pre-trained Bi-LSTM classifier, validated in [31], to predict future activities based on the forecasted signals. The principal contributions of the present work are as follows. First, we demonstrate the feasibility of the proposed HAP system by forecasting and classifying IMU activity signals up to 2.56 s into the future. Our HAP system achieves an average accuracy and precision in activity prediction of 97.96% and 97.92%. These results open up new potential in applications such as fall detection, healthcare assistance, sports monitoring, and more areas where activity prediction could play a critical role. Second, we have proposed a deep learning forecaster based on the Seq2Seq, MA, and PE structures to predict future activity signals. We have demonstrated the forecaster’s capability by comparing it against the conventional forecasters, outperforming them by a minimum of 31.90% in terms of RMSE error and correlation coefficient.

The remainder of this paper is organized as follows. Section 2 introduces the methodology of our proposed HAP system. Section 3 describes the experimental and validation results of the forecaster and classifier of the HAP structure. Section 4 presents the discussion of our work against some prior works and lists possible applications and future works. Finally, Section 5 concludes our work.

## 2. Methodology

**Figure 1:**
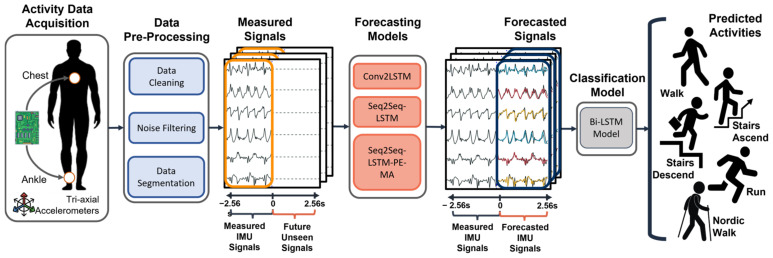
The framework of our proposed HAP system.

Figure 1 illustrates the framework of our proposed HAP system. First, activity signals are measured from two tri-axial IMUs at the chest and ankle of the subjects. The activity signals are then preprocessed and fed into the forecasting models. Finally, the output of the forecasters (i.e., forecasted activity signals) are fed into the pre-trained classifier to predict future activities such as walk, run, Nordic walk, stairs ascend, and stairs descend. The following subsections present the details of each process, including the preprocessing steps of the IMU signals, the forecaster models, and the activity classifier.

### 2.1. IMU Dataset

We conducted our work on a public benchmark dataset for activity recognition in the Physical Activity Monitoring Dataset (PAMAP2) [32]. This database contains IMU multivariate time series data of various activities. It comprises 18 daily activity records collected from nine subjects: one female and eight males, with an average age of 27.22±3.31 years. Four types of sensors were utilized in the dataset, including one temperature sensor and three IMU modules positioned on the subject’s arms, chest, and ankles, recorded at a sampling rate of 100 Hz. Each IMU contains a set of the tri-axial gyroscope (rad/s), magnetometer (μT), and accelerometer (${\mathrm{ms}}^{2}$) sensors with a resolution of 13 bits and scales of ±6 g and ±16 g. Each activity was recorded continuously using wireless sensors, containing missing and erroneous values due to data dropping, loss of communication, hardware setup errors, and system crashes. We have applied a set of preprocessing steps to the IMU signals to remove and clean them, as described in the following subsection.

We have selected five daily activities (i.e., walk, run, Nordic walk, stairs ascend, and descend) from five subjects (i.e., 101, 102, 105, 106, and 107) to train, validate, and test the forecasters and classifier models. For each subject, two tri-axial accelerometers from the chest and ankle were selected, resulting in six channels of motion time series data with a scale of ±16 g. To evaluate the proposed HAP system, a total of 195 min of record for all the subjects are used to obtain the training data of the forecaster, resulting in 2283 activity epochs with a time length of 5.12 s. Then, 39 min of unseen records are used to test data, resulting in 456 epochs. Half of each epoch is used to train the forecasters and the remaining half to test them. Thus, the forecaster is expected to produce future activity signals based on the first half of the epoch. In the classifier’s training, the second half of each epoch is used to produce the label of each activity. The classifier is expected to produce a correct activity label based on the forecasted signals as input.

### 2.2. Preprocessing Steps

A set of preprocessing steps has been applied to the activity data before training the forecast models. First, for data cleaning, the dropout data technique is applied to remove the first and last 35 s of data records, NaN values, and outlier sections by dropping out and replacing them with the average signal value. Second, a low-pass filter with a cutoff frequency of 15 Hz is applied to each channel to remove noise, as performed in [33]. Third, the mean values are removed, and global normalization is applied to limit the signal data between −1 and 1; after this step, a moving average filter of five points is used to filter and obtain a smooth signal. Subsequently, a window overlap sliding technique [34] is used with the five activities to balance and augment the data of each activity. Finally, data segmentation in epochs of 5.12 s for all the accelerometer channels is carried out to obtain the signal needed as input for the deep learning forecasters.

### 2.3. Forecasting IMU Activity Signals

**Figure 2:**
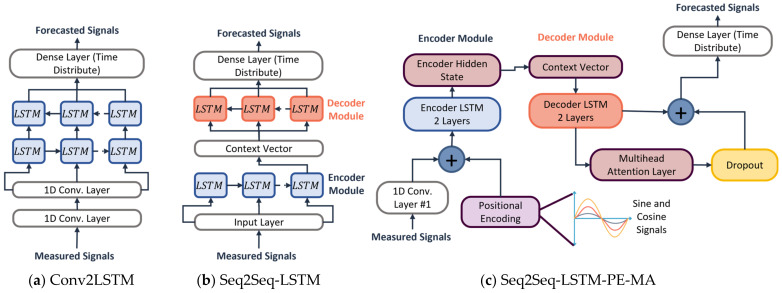
Deep Learning Forecasters: (**a**) Hybrid CNN-to-RNN (Conv2LSTM), (**b**) Sequence-to-Sequence LSTM (Seq2Seq-LSTM), (**c**) Sequence-to-Sequence with Attention and Positional Encoding (Seq2Seq-LSTM-PE-MA).

We have implemented three forecasting models in the proposed HAP system, as shown in Figure 2. Three multivariate deep learning models for signal forecasting use the past IMU records to forecast the unseen signals of six channels. The first model is a hybrid convolution-to-recurrent network named Conv2LSTM. The second model uses a Sequence-to-Sequence LSTM structure named Seq2Seq-LSTM. Finally, our proposed model uses a double-layer Seq2Seq-LSTM structure with MA and PE layers named Seq2Seq-LSTM-PE-MA. The improvements to the third model are based on multi-head attention layers of the base transformer model, which has been successfully used for Natural Language Processing (NLP) [35]. Additionally, we adapted and trained two extra GAN-based models, namely SynSigGAN [26] and TTS-GAN [30]. Detailed descriptions of each model are given in the following subsections.

#### 2.3.1. Conv2LSTM

The first deep learning forecaster is shown in Figure 2, which uses a model composed of CNN and RNN layers based on the structure proposed in [36]. Initially, the measured signals are given as input for two CNN layers to extract relevant features from the IMU activity signals. Subsequently, the obtained features pass through two RNN layers of Long-Short-Term Memory (LSTM) units to capture the temporal dependencies and patterns in the signal over time, giving the model data sequence information of each channel. Using this structure, the model learns and retains significant activity features from the signal extracted in the CNN layers. Regularization is applied with a dropout layer to prevent overfitting. Finally, the last time distributed dense layer is used to process the predicted output using the hidden state results from the last LSTM layer.

The structure of the Conv2LSTM module is presented in Figure 2a and is composed of two 1D convolutional layers with 32 output filters, a kernel size of six, and a ReLU activation function. In sequence, with two LSTM layers of 256 output units and a hyperbolic tangent function. The behavior of the CNN layers is presented in Equation (1).

(1) $$h_{t}^{\left(i\right)}=f\left(\overset{k-1}{\underset{j=0}{\sum }}w_{j}^{\left(i\right)}x_{t+j}+b^{\left(i\right)}\right)$$

where $h_{t}^{\left(i\right)}$ is the output at time step t of the i-th convolutional layers. f is a nonlinear activation function, in our case ReLU. $w_{j}^{\left(i\right)}$ is the weight for the j-th filter in the i-th convolutional layer. $x_{t+j}$ is the input at the current time step. $b^{\left(i\right)}$ is the bias term for the i-th convolutional layer. Finally, k represents the size of the filter. Meanwhile, for the RNN layers, Equation (2) represents the final output equation.

(2) $$h_{t}^{\left(i\right)}=\sigma \left(W_{o}x_{t}+U_{o}h_{t-1}+b_{o}\right)*\tanh\left(f_{t}c_{t-1}+i_{t}g_{t}\right)$$

where $U_{0},{\;W}_{0}$ are the output gate weights. $b_{0}$ the bias value. $c_{t}$ is the cell state value at time t. $i_{t}$ is the input value. $g_{t}$ is the input modulation gate. Finally, a dropout rate of 0.5 is applied before the last convolutional layer with an output dimension of 256.

#### 2.3.2. Seq2Seq-LSTM

Seq2Seq models [37] are proposed as an effective technique in multiple applications involving time series data forecasting [5,38]. These structures are commonly constituted by an encoder–decoder layer implemented with RNN units such as LSTM or GRU. This work uses a Seq2Seq framework composed of LSTM units for multivariate IMU data forecasting. In this model, the encoder processes the past signal time steps individually, producing a sequence of hidden states in which the last one summarizes the observed input data. This information is then stored in a fixed-length context vector alongside a unique start-of-sequence token (the last value of the input for data forecasting) to be used as input for the decoder. Subsequently, the decoder generates the output predicted signal using the context vector and start token. The predicted output of each step is then fed back to the next RNN unit; this process is repeated until the entire forecast output sequence length is completed.

The Seq2Seq-LSTM model is shown in Figure 2b. It comprises an encoder–decoder structure of one RNN layer with 100 LSTM units and a hyperbolic tangent as an activation function. Between the encoder and decoder, a repeat vector that works as one 1D memory layer is added to store the hidden state from the last LSTM unit of the encoder module, preserving the input features. Equation (3) represents the behavior of the module, where $h_{t-1}$ represents the last hidden state, $x_{t}$ the current time series value, t−1 the prior time step value, and Z the fixed-length context vector obtained. The decoder model is represented in the following Equation (4).

(3) $$h_{t}=f_{\mathrm{enc}.}\left(x_{t},h_{t-1}\right)\;\&\;Z=h_{t}$$

(4) $${h^{\prime }}_{t}=f_{\mathrm{dec}.}\left(y_{t-1},h_{t-1}^{\prime },Z\right)\;\&\;\hat{y}=h_{t}^{\prime }W+b$$

where $h_{t}^{\prime }$ represents the last hidden state, Z the encoder output, $y_{t-1}$ and $h_{t-1}^{\prime }$ the previous output and hidden state at time t, respectively, and $\hat{y}$ is the final forecasted output.

#### 2.3.3. Seq2Seq-LSTM-PE-MA

The third model, Seq2Seq-LSTM-PE-MA, is illustrated in Figure 2c. This model has two main implementations from the transformer baseline model the PE and MA layers. This structure has been used in transformer models in recent years for multiple applications due to the ability to parallelize the training and inference process, capture complex patterns, and include long-term dependencies [39]. This model uses the attention mechanism of the PE and MA layers to improve the forecast performance of time series sensor data. The PE module is located at the bottom of the encoder structure. It captures the sequential order of the input time series data providing a notion of data position to the model. This enhancement is achieved by adding the output of this module as a fixed vector to the input time series data previously resized by a 1D convolutional layer. The structure for the PE module is based on the traditional transformer structure made by sine and cosine functions described in Equation (5) to generate the position sequence.

(5) $${\mathrm{PE}}_{\left(\mathrm{pos},2i\right)}=\sin\left(\frac{\mathrm{pos}}{{10,000}^{\frac{2i}{d_{\mathrm{mod}}}}}\right)\;\&{\;\mathrm{PE}}_{\left(\mathrm{pos},2i+1\right)}=\cos\left(\frac{\mathrm{pos}}{{10,000}^{\frac{2i}{d_{\mathrm{mod}}}}}\right)$$

where PE represents the positional encoding. pos is the position. $d_{\mathrm{mod}}$ is the dimension of the embedding input. i is the dimension. With these equations, the wave forms a geometric progression from 2π to 10,000∗2π. This selection follows the criteria made in [40]. The next improvement is the addition of one RNN layer with LSTM units that returns the full sequence of outputs to the second layer on the encoder and decoder modules.

The second transformer model adaptation is based on the MA layer located after the decoder module. This layer performs a self-attention dot product calculation in multiple heads, simultaneously allowing to attend different time series data sections. In this model, we use 16 heads for the attention calculation. A scaled dot-product attention is performed and calculated on each head using Equation (6).

(6) $$\mathrm{Attention}\left(Q,K,V\right)=\mathrm{softmax}\left(\frac{{\mathrm{QK}}^{T}}{\sqrt{d_{k}}\;}\right)V$$

where Q, K, and V represent the queries, keys, and values that are parameters obtained from the input data. $d_{k}$ is the dimension of the keys. This output indicates the priority weights of each value vector and head after being concatenated, regularized, and linearly transformed. The obtained attention is finally added to the decoder’s forecasted signal output. This process increases the correlation between the forecasted and input signal, improving the future classification task by preserving the signal features using the feature position knowledge during the training.

#### 2.3.4. SynSigGAN

The framework SynSigGAN was initially proposed as a data augmentation model for univariable time series applications that involve biosignal data such as the electrocardiogram (ECG), electromyography (EMG), and photoplethysmography (PPG) [26]. This model proposed a general GAN structure using a bidirectional grid LSTM network (BiGridLSTM) of two layers as the generator and a standard CNN network of three layers for the critic network. The objective of the generator module is to create a new signal by utilizing an initial random latent space representation of the original data, with the intention of generating synthetic data that closely resembles the ground truth sample. Conversely, the critic network acts as a binary classifier to determine if the data generated is real or fake. Therefore, BiGridLSTM aims to minimize the $\log(1-D\left(G\left(z\right)\right)$ and the value function expressed in Equation (7) where D and G represent the critic and generator network, respectively.

(7) $$\underset{G}{\min}\;\underset{D}{\max}\;F_{v}\left(D,G\right)={Ε}_{x\sim p_{d}\left(x\right)}\left[\log D\left(x\right)\right]+{Ε}_{z\sim p_{z}\left(z\right)}\left[\log\left(1-D\left(G\left(z\right)\right)\right)\right]$$

#### 2.3.5. TTS-GAN

The TTS-GAN framework was initially used in data augmentation and forecasting works to create synthetic time series data [30]. This model proposes a general GAN structure using the base encoder Transformer module to replace the generator and critic network composed of two main blocks. The first block comprises a multi-head self-attention layer with an embedding size of 100 and five parallel attention heads. Then, the second block uses a feed-forward MLP with a GELU activation function. Both blocks use a normalization, dropout layer, and residual connection to prevent the model overfitting and vanishing problem. Finally, a Convolutional 2D layer with a kernel size of (1, 1) is used as the last layer of the generator encoder to reduce and match the data dimension of the predicted result with the ground truth samples. This model uses the Mean Square Error (MSE) loss to update the parameters during the generator and the critic network training as represented in Equations (8) and (9).

(8) $$D_{\mathrm{loss}}=\mathrm{MSE}\left(D\left(\mathrm{gt}\right),{\mathrm{gt}}_{\mathrm{lbl}}\right)+\mathrm{MSE}\left(D\left(G\left(z\right)\right),{\mathrm{pred}}_{\mathrm{lbl}}\right)$$

(9) $$G_{\mathrm{loss}}=\mathrm{MSE}\left(D\left(G\left(z\right)\right),{\mathrm{gt}}_{\mathrm{lbl}}\right)$$

where $D_{\mathrm{loss}}{\;\mathrm{and}\;G}_{\mathrm{loss}}$ represent the loss value to minimize in the critic and generator network. $D\left(x\right)$ is the decision output of the critic network. $G\left(z\right)$ is the predicted signal of the generator. gt the ground truth signal and ${\mathrm{gt}}_{\mathrm{lbl}}{\;\mathrm{and}\;\mathrm{pred}}_{\mathrm{lbl}}$ are the labels of the ground truth and predicted signals, respectively.

### 2.4. Bi-LSTM Network for Activity Classification

**Figure 3:**
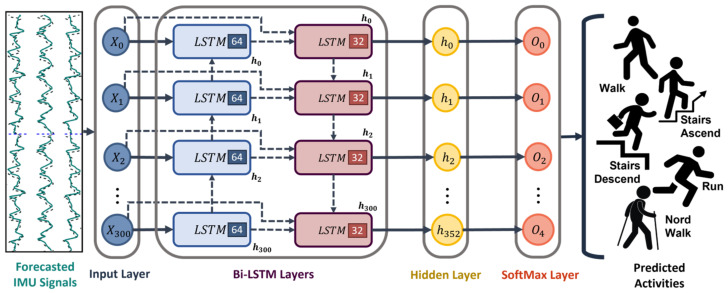
Bi-LSTM classifier used for activity prediction.

In order to recognize future activities using the signals obtained from the forecast models, we used a Bidirectional Long Short-Term Memory (Bi-LSTM) model. The classification model proposed in this work is detailed in [31]. This model has been proven to obtain high classification accuracy in many prior HAR applications. Figure 3 shows the model structure composed of two principal components: the Bi-LSTM layers and a fully connected layer with a Softmax as an activation function.

The classifier is designed to capture the temporal dependencies of the input data by processing in both forward and backward directions considering the past and future context data. Meanwhile, the full connection layer produces a probability distribution over the five proposed activities by giving the highest probability to the predicted activity. Equation (10) represents the Bi-LSTM operation.

(10) $$h_{t}^{f}={\mathrm{LSTM}}_{f}\left(x_{t},h_{t-1}^{f},c_{t-1}^{f}\right);{\;h}_{t}^{b}={\mathrm{LSTM}}_{b}\left(x_{t},h_{t-1}^{b},c_{t-1}^{b}\right)\to {\;h}_{\mathrm{total}}=\left[h_{t}^{f},h_{t}^{b}\right]$$

where ${\mathrm{LSTM}}_{f}{\;\mathrm{and}\;\mathrm{LSTM}}_{b}$ represent the forward and backward layers. $x_{t}$ is the input sequence at the time step t. $h_{t}^{f},{\;\mathrm{and}\;h}_{t}^{b}$ are the hidden state for the forward and backward layers, and $c_{t}^{f}{\;\mathrm{and}\;c}_{t}^{b}$ are the cell states. The final output $h_{\mathrm{total}}$ represents the concatenated values of both layers. The classifier model contains two Bi-LSTM layers with 64 and 32 units, a subsequent ReLU activation function, a hidden layer with 352 neurons, and one SoftMax layer with 5 output neurons.

## 3. Experiment Results

The following subsections detail the training procedures for both the predictor and classifier models and describe the evaluation and performance metrics for both sections. Finally, the experimental results for HAP are presented.

### 3.1. Training and Evaluation Procedures

The forecaster models were trained using 2283 epochs of 5.12 s for each subject in the dataset. The first half of 2.56 s from the entire epoch was used as input during the forecaster training procedure. Meanwhile, the second half of the epoch as the training target was used to evaluate the model performance by comparing the forecasted output against the ground truth signals (i.e., new target signals). All the forecaster models were trained for 240 iterations with a batch size of 25 using the Adams optimizer and mean square error as a loss function with a learning rate of 0.0003 to ensure fast convergence.

In contrast, the Bi-LSTM classifier model was trained using a set of 2283 epochs using the second half of the training epochs to match the size of the forecasted output data. During the training, the Adams algorithm as an optimizer and Categorical Cross Entropy as a loss function across 240 epochs were used with a batch size of 10 and a learning rate of 0.0005. First, we used 456 unseen measurement epochs to obtain the reference activity label. In the second step, we used 289 epoch results from each forecaster as input to obtain the corresponding predicted activity labels. The training process for both models was conducted in a computer with an Nvidia RTX 2070 GPU of 8 Gb of VRAM memory using Python 3.8 with Tensorflow and Keras libraries.

### 3.2. Evaluation Metrics

In order to assess the performance of the forecast models, we selected two evaluation metrics to compare the model’s output with the ground truth IMU signals [21,23,41]. These metrics are the root mean square error (RMSE) and correlation coefficient (CORR). Using these metrics, we have determined the accuracy of the forecaster models in predicting future IMU signals. The RMSE measures the average squared differences between the ground truth and the forecasted signals providing information about the average deviation between the signals; this metric is calculated with Equation (11).

The correlation coefficient CORR measures the strength of the linear relationship between the signals by being sensitive to the trend difference. The coefficient ranges from −1 to+1, where a value of ±1 suggests a perfect correlation, and as the value goes toward 0, the correlation weakens. The sign of the coefficient indicates if there is a positive or negative relation. This metric is described in Equation (12).

(11) $$\mathrm{RMSE}=\sqrt{\frac{1}{n}\overset{n}{\underset{i=1}{\sum }}{\left(y_{i}-\hat{y_{i}}\right)}^{2}}$$

(12) $$\mathrm{CORR}=\frac{\overset{n}{\underset{i=1}{\sum }}\left(y_{i}-\bar{y}\right)({\hat{y}}_{i}-\bar{\hat{y}})}{\sqrt{\overset{n}{\underset{i=1}{\sum }}{\left(y_{i}-\bar{y}\right)}^{2}\overset{n}{\underset{i=1}{\sum }}(\hat{y_{i}}-\bar{\hat{y}}{)}^{2}}}$$

where n is the number of observations of the forecast signal. $y_{i}$ are the ground truth values of the ith observation in the signal. ${\hat{y}}_{i}$ are the predicted values of the ith observation. $\bar{y}$ is the mean of the ground truth data. $\bar{\hat{y}}$ is the mean of the signal-predicted values.

For the evaluation of the Bi-LSTM classifier, three standard metrics, accuracy, precision, and F1-score, are used. The accuracy measures the total percentage of correct positive and negative predicted labels; meanwhile, the precision indicates the truly positive predictions made by the model among the positive predictions. Finally, the F1-score indicates the harmonic mean between the precision and recall providing a balanced assessment of the model performance. We use the classification results divided into true positive predictions. $T_{p}$, true negatives, $T_{n}$, false positives, $F_{p}$, and false negatives $F_{n}$ to calculate these metrics using the formulas described in Equation (13). Through these metrics, we compare the performance of the HAP system using ground truth data or each forecasted signal as input of the classifier.

(13) $$\mathrm{Acc}.=\frac{T_{p}+T_{n}}{F_{p}+F_{n}+T_{p}+T_{n}}\;;\mathrm{Prec}=\frac{T_{p}}{F_{p}+T_{p}};\;F1-\mathrm{score}=\frac{T_{p}}{T_{p}+\frac{1}{2}\left(F_{p}+F_{n}\right)}$$

### 3.3. IMU Signal Forecaster Performance

Table 1 shows the five forecasters performances in terms of RMSE and CORR results obtained from Subject #101. Performance improvement is observed from SynSigGAN to the proposed framework Seq2Seq-LSTM-PE-MA across all the activities. Regarding average RMSE from the activities, the proposed Seq2Seq-LSTM-PE-MA model improved by 68.73% with respect to the SynSigGAN model, 63.24% to Conv2LSTM, 53.75% to the base Seq2Seq-LSTM model, and 31.90% against the TTS-GAN model. Moreover, concerning the average CORR, the proposed model improves by 36.46% to the SynSigGAN, 28.87% to Conv2LSTM, 19.74% to the Seq2SeqLSTM, and 8.30% against the TTS-GAN model.

Considering the highest RMSE and lowest CORR results for each model in the most challenging activity of stairs descend, the SynSigGAN model showed the highest RMSE and lowest CORR with values of 0.366 and 0.566, respectively. These values are related to the SynSigGAN model’s single-channel design, which degrades the forecast across the rest of the channels. A slight improvement is achieved with the Conv2LSTM and the Seq2Seq-LSTM model, with an RMSE of 0.336 and 0.274 and a CORR of 0.607 and 0.691, respectively. More consistent results were obtained with the two models that involve a transformer attention module, with an RMSE and CORR of 0.176 and 0.823 for the TTS-GAN model. Finally, our proposed network overcame this prior GAN model obtaining an RMSE value of 0.114 and a CORR percentage of 0.907. Our framework shows a clear performance improvement against traditional deep learning models such as the Conv2LSTM and Seq2Seq-LSTM and even the recent TTS-GAN model.

**Figure 4:**
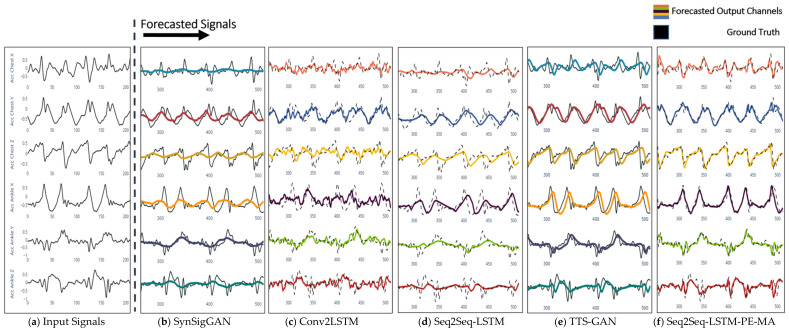
Forecasted signals of the Nordic walk activity by (**b**) SynSigGAN, (**c**) Conv2LSTM, (**d**) Seq2Seq-LSTM, (**e**) TTS-GAN, and (**f**) Seq2Seq-LSTM-PE-MA using the (**a**) Input Past Signals.

Figure 4 illustrates the forecasting results of the actual multichannel signals for the activity of the Nordic walk. Figure 4a shows the input for all the models. Figure 4b illustrates the forecasting result for the SynSigGAN model, where forecasted multichannel signals in color are superimposed over the ground truth signals with a notable absence of peak values. Figure 4c shows the Conv2LSTM model failing to predict the trends and peak values of the signal. Although this model achieves better results against SynSigGAN, reaching an RMSE of 0.278 and a CORR of 0.685, the noise degenerates the output signals, making it harder to interpret. Figure 4d shows the Seq2Seq-LSTM output signal, showing less noise and predicting the signal features with an RMSE error of 0.219 and a CORR of 0.765. Despite the patterns located at the end of the window being kept, peak features at the beginning section are lost, creating a flat signal at the beginning of each epoch due to the fixed attention size of the context layer in the Seq2Seq-LSTM model. Figure 4e shows the TTS-GAN output, where the forecasted signals make prediction improvements against the prior models by correctly predicting the signal patterns and features. However, the model still presents difficulties, such as the absence of high-frequency components in some channels. Finally, Figure 4e shows the Seq2Seq-LSTM-PE-MA model results with an RMSE of 0.096 and CORR of 0.931. This model produces the best prediction by matching the ground truth signals due to the positional encoding and the multi-head attention layers.

**Figure 5:**
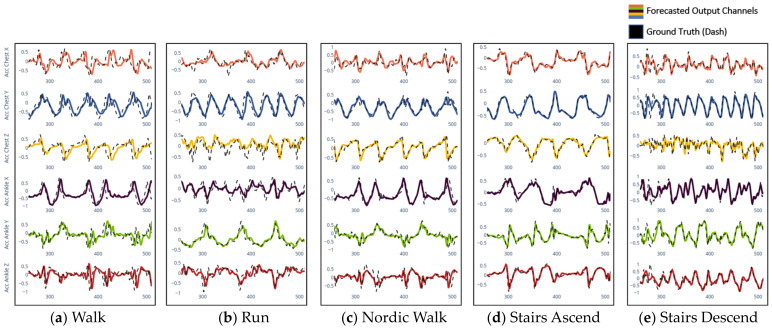
Forecasted signals for all five activities by the Seq2Seq-LSTM-PE-MA model.

To demonstrate the performance of the proposed forecaster, we have used the same epoch input of 2.56 s from Subject #101 to obtain the predicted signals across all the selected daily activities, as shown in Figure 5. The proposed model shows its capacity by predicting the signal patterns and relevant features from the signal, such as those shown in the activities of run and stairs descent. In addition, the model presents the capacity to predict all the peak values across all the channels of the IMU sensor compared to the forecast models that do not present a multi-head attention mechanism. This capacity allows to differentiate between each activity channel, making it possible to predict future activities precisely.

Table 2 shows the forecasting performance of the proposed Seq2Seq-LSTM-PE-MA from the five subjects. The average result for each activity shows that the maximum RMSE error of 0.127 ± 0.029 is obtained in the run activity with a CORR of 0.888 ± 0.039. In contrast, the activity of stairs ascend has the best prediction among the subjects, with a total CORR of 0.913 ± 0.007 and an RMSE of 0.109 ± 0.005. Finally, the rest of the activities across all the subjects show similar performance, reaching an overall mean and standard deviation for RMSE error of 0.118 ± 0.006 and a CORR of 0.901 ± 0.009.

### 3.4. HAP Performance

**Figure 6:**
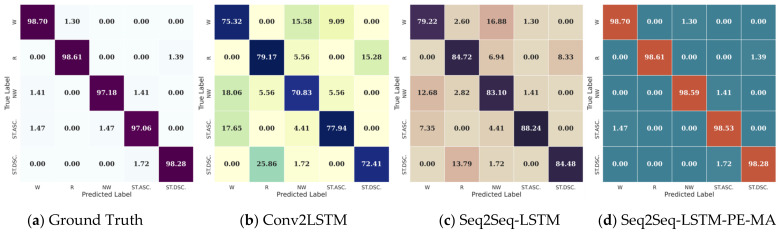
Sample confusion matrices results of HAP for walk (W), run (R), Nordic walk (NW), stairs ascend (STASC), and stairs descend (STDSC) using: (**a**) Ground Truth data and the forecasted activity data from (**b**) Conv2LSTM, (**c**) Seq2Seq-LSTM, and (**d**) Seq2Seq-LSTM-PE-MA.

The proposed HAP model is tested through the Bi-LSTM model with the forecasted epochs of 2.56 s. Figure 6 shows a sample of confusion matrices for the five activities of Subject #101. Figure 6a shows the confusion matrix obtained using the ground truth measured IMU motion signal as input for the Bi-LSTM module, where all the activities show an accuracy higher than 98.29%. Figure 6b,c present the sample confusion matrices of the Bi-LSTM classifier using as input the result from three of the five baseline forecasters used in this work, the Conv2LSTM, the Seq2Seq-LSTM, and the proposed Seq2Seq-LSTM-PE-MA model, respectively. Regarding that, both models obtained an accuracy of 75.216% and 83.81%; misclassification still occurred for certain activities such as Nordic walk and walk, stairs descend, and run due to the resemblance in signal data among each activity. Therefore, most confusions are mainly due to errors in the forecasted signals and classifier capacity. As a result, the Conv2LSTM and Seq2Seq-LSTM forecaster cannot predict the signals with their representative features generating similar signals on different channels, creating confusion and hindering the classification. In addition, compared to the prior results, the SynSigaGAN forecaster achieved the lowest accuracy prediction of all the models reaching only 57.121%. Meanwhile, the TTS-GAN structure outperformed the Conv2LSTM and the Seq2Seq-LSTM models, reaching a maximum accuracy of 89.091%.

In contrast, Figure 6d shows the confusion matrix result for the proposed model Seq2Seq-LSTM-PE-MA, where an average accuracy of 97.96% is achieved. Finally, in terms of accuracy, the proposed model shows an improved performance of 41.69% with respect to the SynSigGAN model, 23.21% to the Conv2LSTM, 14.44% to the Seq2Seq-LSTM, and 9.053% against the TTS-GAN model. Therefore, the Seq2Seq-LSTM-PE-MA model is the unique structure capable of obtaining results over 97% and reaching a similar performance using the ground truth signal as input.

The overall performance of HAP with the proposed prediction system across the five subjects is summarized in Table 3. This table compares the obtained result from the BILSTM classifier using the forecasted signals and ground truth data as input. The overall accuracy, precision, and F1-score performance are summarized. Across the five subjects, the minimum accuracy reached was 97.698% for subject #102, with precision and an F1-score of 97.599% and 97.953%, respectively. Meanwhile, the maximum accuracy of 98.221% was achieved, with subject #105 reaching a precision of 98.145% and an F1-score of 98.109%. As a result, the proposed HAP framework achieves an average accuracy, precision, and F1-score of 97.96% ± 0.228, 98.14% ± 0.247, and 97.87% ± 0.316 reaching a minimum difference of 0.33%, 0.23%, and 0.26% compared with the results using the ground truth signal as input.

## 4. Discussion

This paper presents a novel HAP system based on the forecasted IMU activity signals from two tri-axial accelerometers in the subject’s chest and left ankle. The proposed HAP system is composed of a time series forecaster and a pre-trained classifier. A novel deep learning forecaster is built using a Seq2Seq-LSTM model with MA and PE layers as attention mechanisms. Meanwhile, a Bi-LSTM model is used as a classifier to label the predicted activities based on the forecasted motion signals.

Regarding forecasting of IMU activity signals, the proposed Seq2Seq-LSTM-PE-MA model was compared against four state-of-art time series forecasters, SynSigGAN, Conv2LSTM, Seq2Seq-LSTM, and TTS-GAN, as depicted in Figure 4. The proposed Seq2Seq-LSTM-PE-MA model achieved the best results in forecasting the signals with an RMSE error under 0.111 and a CORR over 0.916 between the ground truth and forecasted signals for all tested activities. Similar works in forecasting time series signals have used RNN and transformer variations to improve model retention. In [21], a neural network mixed with Fourier transform was used, achieving an RMSE value of 0.134, predicting a total of 0.6 s ahead using 1 s of input data. Meanwhile, in [22], an adversarial training based on a transformer Generator, a CNN Critic Network, and an action classifier achieved an RMSE value of 0.180 between the forecast and ground truth signals in fatigue prediction future 0.8 s. Compared to these works, our model can handle multivariate signal forecasting signals up to 2.56 s compared to prior studies. Due to the majority of prior works for time series, forecasters are used in applications such as weather, asset prediction, or data augmentation, and the lack of forecasters used for human activity prediction is not possible to directly compare prior models with the structure proposed in this work. Therefore, we have added two forecasters based on GAN networks, the SynSigGAN [26] and the TTS-GAN [30] model. We retrained and tested these models using the five subjects from the PAMAP2 dataset using the same input of 2.56 s, obtaining an average RMSE error of 0.351 and 0.169 for the SynSigGAN and TTS-GAN, respectively. In contrast to the added GAN models, our approach does not have the inherent drawbacks of the GAN networks, such as mode collapse and training instability. In terms of average RMSE, our model achieved a value of 0.111, obtaining an improvement of 17.16%, 38.33%, 56.87%, and 35.55% compared to the two related models and the adapted GAN models, respectively.

Regarding human activity prediction in the proposed system, an average accuracy and precision of 97.961% and 97.920% were achieved using the forecasted signals, reaching a minimum difference of 0.329% and 0.230% compared to the classification results using the ground truth data. Concerning HAP performance, prior attempts have shown results in predicting future actions based on a long signal record using attention layers. However, few works have been used for multiclass classification, especially for daily activity tasks. In [43], activity prediction and action transition are presented using a self-recorded dataset reaching a precision and F1-score of 95.0% and 97.8% in daily activities such as walking and running. Although this model could obtain intention prediction results, it is related to the complete sequence of actions followed by the subject and not only the current activity. In [22], a multiclass fatigue prediction up to 0.8 s is carried out using the forecasted IMU signals, achieving an average accuracy of 83% with a correlation coefficient of 92%. Compared to these prior works, our HAP system only uses 2.56 s of IMU data instead of the hold record to perform the activity prediction, achieving an average accuracy of 97.981% and a precision improvement of 3.13%.

Despite the importance of HAP, most prior works have focused only on videos as input data instead of time series sensors. Prior video-based HAR works based on adversarial learning and recurrent network models such as Rolling-LSTM, HARD-Net, and CGAN achieved accuracies of 83.5%, 87.54%, and 80.9%, respectively, for daily activities such as washing the dishes, moping the floor, or handshaking [14,15,16]. Compared to these video-based systems, our approach can obtain an improved performance of at least 10.441% of accuracy in predicting future activities. State-of-art HAR systems using the same PAMAP2 dataset [44,45,46] for multiclass classification have shown accuracy results of 93,75%, 94.29%, and 99.64%. Compared to these works, the proposed HAP system can reach an accuracy of 97.96% at predicting future activities up to 2.56 s ahead. The proposed HAP system is open for future improvements. First, additional sensors in various positions, such as the wrist or head, should be considered to recognize more complex activities that use more parts of the body. Second, multi-modal sensors and their prediction should be investigated by involving various sensors in the prediction, such as gyroscopes, temperature, light detectors, or even ECG, since these sensors are invaluable in healthcare monitoring and smart homes assistance to implement more robust and complete prediction applications aimed for healthcare assistance. Third, to generalize the HAP system, studying more activities using different subjects from more datasets is necessary. Finally, optimizing and embedding the proposed HAP model into an edge device will make the HAP system portable and capable of running in real time.

## 5. Conclusions

In this study, we present a novel HAP system based on forecasted IMU activity signals. Our system consists of two main components: (i) a deep Seq2Seq forecaster model based on a transformer multi-head self-attention module and positional encoding layers for IMU motion signal prediction and (ii) a Bi-LSTM model as a deep classifier to label the next activity using the forecasted signals. Using two IMU accelerometer sensors, our HAP system accurately predicts five daily human activities, achieving an accuracy of 97.960%, precision of 97.920%, and F1-Score of 97.870% up to 2.56 s ahead. Our findings across the selected subjects demonstrate the feasibility of implementing a sensor-based HAP system capable of being used with unseen data. This HAP capability holds significant importance for applications that require an early or anticipated response, such as fall detection, healthcare monitoring, sports performance monitoring, assistive robots in industrial or rehabilitation, and smart home assistance.

## Figures and Tables

**Table 1:** Evaluation results of the forecasters with Subject #101.

Models	Forecast Evaluation Metrics (Subject #101)											
	Walk		Run		Nordic Walk		Stairs Ascend		Stairs Descend		Mean	
	RMSE	CORR	RMSE	CORR	RMSE	CORR	RMSE	CORR	RMSE	CORR	RMSE	CORR
SynSigGAN [26]	0.355	0.581	0.340	0.601	0.342	0.599	0.369	0.563	0.366	0.566	0.355	0.582
Conv2LSTM [36]	0.269	0.697	0.297	0.659	0.278	0.685	0.330	0.615	0.336	0.607	0.302	0.653
Seq2Seq-LSTM [42]	0.205	0.784	0.244	0.731	0.219	0.765	0.257	0.714	0.274	0.691	0.240	0.737
TTS-GAN [30]	0.180	0.817	0.155	0.851	0.160	0.844	0.145	0.865	0.176	0.823	0.163	0.840
Seq2Seq-LSTM-PE-MA	0.110	0.912	0.105	0.919	0.096	0.931	0.111	0.911	0.114	0.907	0.111	0.916

**Table 2:** Activity signal forecasting results by the Seq2Seq-LSTM-PE-MA model from the five subjects.

Selected Subjects	Seq2Seq-LSTM-PE-MA									
	Walk		Run		Nordic Walk		Stairs Ascend		Stairs Descend	
	RMSE	CORR	RMSE	CORR	RMSE	CORR	RMSE	CORR	RMSE	CORR
#101	0.110	0.912	0.105	0.919	0.096	0.931	0.111	0.911	0.114	0.907
#102	0.118	0.901	0.177	0.822	0.133	0.881	0.106	0.917	0.125	0.891
#105	0.142	0.868	0.107	0.916	0.099	0.927	0.102	0.923	0.096	0.931
#106	0.132	0.882	0.122	0.896	0.123	0.894	0.111	0.911	0.122	0.895
#107	0.111	0.910	0.127	0.889	0.117	0.903	0.116	0.904	0.105	0.918
Mean ± SD	0.123 ± 0.014	0.895 ± 0.019	0.127 ± 0.029	0.888 ± 0.039	0.113 ± 0.016	0.907 ± 0.021	0.109 ± 0.005	0.913 ± 0.007	0.113 ± 0.012	0.908 ± 0.016

**Table 3:** Evaluation of the HAP results using the forecasted data of the proposed model for the five selected subjects against the ground truth data.

Selected Subjects	Seq2Seq-LSTM-PE-MA Subject Results					
	Accuracy (%)		Precision (%)		F1-Score (%)	
	Model Result	Ground Truth	Model Result	Ground Truth	Model Result	Ground Truth
#101	97.981	98.125	97.969	98.012	97.953	97.975
#102	97.698	97.901	97.599	97.992	97.953	97.975
#105	98.291	98.340	98.145	98.028	98.109	98.350
#106	97.799	98.193	97.725	98.014	97.315	98.134
#107	98.025	98.903	98.145	98.714	98.015	98.204
Mean ± SD	97.96 ± 0.228	98.29 ± 0.376	97.92 ± 0.247	98.15 ± 0.314	97.87 ± 0.316	98.13 ± 0.160

## Data Availability

Not applicable.

## References

[1] Tang Y., Zhang L., Wu H., He J., Song A. (2022). Dual-Branch Interactive Networks on Multichannel Time Series for Human Activity Recognition. IEEE J. Biomed. Health Inform..

[2] Yeh Y.H., Wong D.P.Y., Lee C.T., Chou P.H. (2022). Deep Learning-Based Real-Time Activity Recognition with Multiple Inertial Sensors. IVSP’22: Proceedings of the 2022 4th International Conference on Image, Video and Signal Processing, Singapore, 18–20 March 2022.

[3] Ishwarya K., Alice Nithya A. (2022). Performance-Enhanced Real-Time Lifestyle Tracking Model Based on Human Activity Recognition (PERT-HAR) Model through Smartphones. J. Supercomput..

[4] Huo W., Mohammed S., Moreno J.C., Amirat Y. (2016). Lower Limb Wearable Robots for Assistance and Rehabilitation: A State of the Art. IEEE Syst. J..

[5] Fernandes C., Matos L.M., Folgado D., Nunes M.L., Pereira J.R., Pilastri A., Cortez P. A Deep Learning Approach to Prevent Problematic Movements of Industrial Workers Based on Inertial Sensors. Proceedings of the International Joint Conference on Neural Networks.

[6] Mohamed M., El-Kilany A., El-Tazi N. (2022). Future Activities Prediction Framework in Smart Homes Environment. IEEE Access.

[7] Hussain Z., Sheng Q.Z., Zhang W.E. (2020). A Review and Categorization of Techniques on Device-Free Human Activity Recognition. J. Netw. Comput. Appl..

[8] Balaha H.M., Hassan A.E.S. (2023). Comprehensive Machine and Deep Learning Analysis of Sensor-Based Human Activity Recognition. Neural Comput. Appl..

[9] Hassan M.M., Uddin M.Z., Mohamed A., Almogren A. (2018). A Robust Human Activity Recognition System Using Smartphone Sensors and Deep Learning. Future Gener. Comput. Syst..

[10] Yadav S.K., Tiwari K., Pandey H.M., Akbar S.A. (2021). A Review of Multimodal Human Activity Recognition with Special Emphasis on Classification, Applications, Challenges and Future Directions. Knowl. Based Syst..

[11] Kim Y.W., Joa K.L., Jeong H.Y., Lee S. (2021). Wearable Imu-Based Human Activity Recognition Algorithm for Clinical Balance Assessment Using 1d-Cnn and Gru Ensemble Model. Sensors.

[12] Anagnostis A., Benos L., Tsaopoulos D., Tagarakis A., Tsolakis N., Bochtis D. (2021). Human Activity Recognition through Recurrent Neural Networks for Human–Robot Interaction in Agriculture. Appl. Sci..

[13] Chen K., Zhang D., Yao L., Guo B., Yu Z., Liu Y. (2021). Deep Learning for Sensor-Based Human Activity Recognition: Overview, Challenges, and Opportunities. ACM Comput. Surv..

[14] Li T., Liu J., Zhang W., Duan L. (2020). HARD-Net: Hardness-AwaRe Discrimination Network for 3D Early Activity Prediction. Lecture Notes in Computer Science (Including Subseries Lecture Notes in Artificial Intelligence and Lecture Notes in Bioinformatics).

[15] Xu W., Yu J., Miao Z., Wan L., Ji Q. Prediction-CGAN: Human Action Prediction with Conditional Generative Adversarial Networks. Proceedings of the MM 2019—Proceedings of the 27th ACM International Conference on Multimedia.

[16] Furnari A., Farinella G.M. What Would You Expect? Anticipating Egocentric Actions with Rolling-Unrolling LSTMs and Modality Attention. Proceedings of the ICCV Open Access by Computer Vision Foundation.

[17] Farha Y.A., Richard A., Gall J. When Will You Do What?—Anticipating Temporal Occurrences of Activities. Proceedings of the IEEE Computer Society Conference on Computer Vision and Pattern Recognition.

[18] Weiss G.M., Yoneda K., Hayajneh T. (2019). Smartphone, and Smartwatch-Based Biometrics Using Activities of Daily Living. IEEE Access.

[19] Banos O., Garcia R., Holgado-Terriza J.A., Damas M., Pomares H., Rojas I., Saez A., Villalonga C. (2014). MHealthDroid: A Novel Framework for Agile Development of Mobile Health Applications. Lecture Notes in Computer Science (Including Subseries Lecture Notes in Artificial Intelligence and Lecture Notes in Bioinformatics).

[20] Al-Qaness M.A.A., Dahou A., Elaziz M.A., Helmi A.M. (2023). Multi-ResAtt: Multilevel Residual Network with Attention for Human Activity Recognition Using Wearable Sensors. IEEE Trans Ind. Inf..

[21] Kim T., Park J., Lee J., Park J. (2021). Predicting Human Motion Signals Using Modern Deep Learning Techniques and Smartphone Sensors. Sensors.

[22] Jiang Y., Malliaras P., Chen B., Kulić D. (2022). Real-Time Forecasting of Exercise-Induced Fatigue from Wearable Sensors. Comput. Biol. Med..

[23] Soleimani R., Lobaton E. (2022). Enhancing Inference on Physiological and Kinematic Periodic Signals via Phase-Based Interpretability and Multi-Task Learning. Information.

[24] Shih S.Y., Sun F.K., Lee H.Y. (2019). Temporal Pattern Attention for Multivariate Time Series Forecasting. Mach. Learn..

[25] Brophy E., Wang Z., She Q., Ward T. (2023). Generative Adversarial Networks in Time Series: A Systematic Literature Review. ACM Comput. Surv..

[26] Hazra D., Byun Y.C. (2020). SynSigGAN: Generative Adversarial Networks for Synthetic Biomedical Signal Generation. Biology.

[27] Yoon J., Jarrett D., van der Schaar M. (2019). Time-Series Generative Adversarial Networks. Adv. Neural. Inf. Process. Syst..

[28] Sumiya Y., Horie K., Shiokawa H., Kitagawa H. Nr-GAN: Noise Reduction GaN for Mice Electroencephalogram Signals. Proceedings of the ICBSP’19: Proceedings of the 2019 4th International Conference on Biomedical Imaging, Signal Processing.

[29] Hyland S.L., Zurich E., Esteban C., Rätsch ETH Zurich G. (2017). Real-Valued (Medical) Time Series Generation with Recurrent Conditional GANs. arXiv.

[30] Li X., Metsis V., Wang H., Ngu A.H.H. (2022). TTS-GAN: A Transformer-Based Time-Series Generative Adversarial Network. Lecture Notes in Computer Science (Including Subseries Lecture Notes in Artificial Intelligence and Lecture Notes in Bioinformatics).

[31] Jaramillo I.E., Jeong J.G., Lopez P.R., Lee C.H., Kang D.Y., Ha T.J., Oh J.H., Jung H., Lee J.H., Lee W.H. (2022). Real-Time Human Activity Recognition with IMU and Encoder Sensors in Wearable Exoskeleton Robot via Deep Learning Networks. Sensors.

[32] Reiss A., Stricker D. Introducing a New Benchmarked Dataset for Activity Monitoring. Proceedings of the International Symposium on Wearable Computers, ISWC.

[33] Luwe Y.J., Lee C.P., Lim K.M. (2022). Wearable Sensor-Based Human Activity Recognition with Hybrid Deep Learning Model. Informatics.

[34] Guennec A.L., Malinowski S., Tavenard R. Data Augmentation for Time Series Classification Using Convolutional Neural Networks. Proceedings of the ECML/PKDD Workshop on Advanced Analytics and Learning on Temporal Data.

[35] Vaswani A., Shazeer N., Parmar N., Uszkoreit J., Jones L., Gomez A.N., Kaiser Ł., Polosukhin I. (2017). Attention Is All You Need. Adv. Neural. Inf. Process. Syst..

[36] Canizo M., Triguero I., Conde A., Onieva E. (2019). Multi-Head CNN–RNN for Multi-Time Series Anomaly Detection: An Industrial Case Study. Neurocomputing.

[37] Sutskever I., Vinyals O., Le Q.V. (2014). Sequence to Sequence Learning with Neural Networks. Adv. Neural Inf. Process. Syst..

[38] Wang X., Cai Z., Luo Y., Wen Z., Ying S. (2022). Long Time Series Deep Forecasting with Multiscale Feature Extraction and Seq2seq Attention Mechanism. Neural Process. Lett..

[39] Wen Q., Zhou T., Zhang C., Chen W., Ma Z., Yan J., Sun L. (2022). Transformers in Time Series: A Survey. arXiv.

[40] Gehring J., Auli M., Grangier D., Yarats D., Dauphin Y.N. (2017). Convolutional Sequence to Sequence Learning. Proceedings of the International Conference on Machine Learning.

[41] Kong Y., Fu Y. (2022). Human Action Recognition and Prediction: A Survey. Int. J. Comput. Vis..

[42] Bahdanau D., Cho K.H., Bengio Y. Neural Machine Translation by Jointly Learning to Align and Translate. Proceedings of the 3rd International Conference on Learning Representations, ICLR 2015—Conference Track Proceedings.

[43] Lee S.H., Lee D.W., Kim M.S. (2023). A Deep Learning-Based Semantic Segmentation Model Using MCNN and Attention Layer for Human Activity Recognition. Sensors.

[44] Tang Y., Zhang L., Min F., He J. (2023). Multiscale Deep Feature Learning for Human Activity Recognition Using Wearable Sensors. IEEE Trans. Ind. Electron..

[45] Challa S.K., Kumar A., Semwal V.B. (2022). A Multibranch CNN-BiLSTM Model for Human Activity Recognition Using Wearable Sensor Data. Vis. Comput..

[46] Kumar P., Suresh S., Tools M. (2023). Deep-HAR: An Ensemble Deep Learning Model for Recognizing the Simple, Complex, and Heterogeneous Human Activities. Multimed. Tools Appl..

